# Cytotoxicity of Esculetin Compared with Vinblastine and Paclitaxel in PC-3 Prostate Cancer Cells †

**DOI:** 10.3390/curroncol32090526

**Published:** 2025-09-20

**Authors:** Ana I. García-Pérez, Virginia Rubio, Angel Herráez, Lilian Puebla, José C. Diez

**Affiliations:** Unidad de Bioquímica y Biología Molecular, Departamento de Biología de Sistemas, Universidad de Alcalá, 28805 Alcalá de Henares, Spain; ana.garcia@uah.es (A.I.G.-P.); tizas_tck@hotmail.com (V.R.); angel.herraez@uah.es (A.H.); lilian.puebla@uah.es (L.P.)

**Keywords:** apoptosis, esculetin, paclitaxel, prostate, vinblastine

## Abstract

Background/Objectives: Metastatic prostate cancer is among the therapy-resistant human neoplasms. PC-3 is a commonly used experimental cell line that does not express androgen receptors. We compared the cytotoxicity of esculetin with that of vinblastine and paclitaxel on prostatic tumour PC-3 cells. Methods: Cells were treated with either esculetin (100 or 250 μM), vinblastine (50 μM) or paclitaxel (100 or 200 μM) for 19 to 72 h. Cells were assessed for metabolic viability, membrane integrity, DNA fragmentation and cell cycle analysis. Apoptosis was checked with annexin and propidium iodide. Results: Esculetin decreased the metabolic activity of PC-3 cells in a time- and concentration-dependent way. The metabolic activity of vinblastine- and paclitaxel-treated cells did not show time-dependence. Cells treated with 250 µM esculetin for 48 or 72 h showed apoptosis levels similar to those produced by 50 µM vinblastine at these incubation times or by 200 µM paclitaxel at 19 h. Vinblastine and paclitaxel produced cell cycle arrest in the G2/M phase after incubation for 19 h. In contrast, esculetin did not significantly affect the cell cycle. Conclusions: A differential action of esculetin on PC-3 prostate cells may be inferred. This may be relevant for novel therapies against resistant prostate cancer.

## Simple Summary

Metastatic prostate cancer is one of the most therapy-resistant human neoplasms. Commonly used chemotherapy drugs for metastatic prostate cancer include paclitaxel and vinblastine. Esculetin is an antioxidant that shows cytotoxic effects on some tumour cells. In this work, we show different cytotoxic effects of the antioxidant esculetin with respect to antimitotic vinblastine and paclitaxel on the human prostatic tumour PC-3 cell line. Esculetin decreased the metabolic activity of PC-3 cells in a time- and concentration-dependent way, whereas vinblastine and paclitaxel did not display such time-dependence. However, PC-3 cells treated with high concentrations of esculetin for long times showed apoptosis levels like those produced by vinblastine in the same times or by paclitaxel at 19 h. Vinblastine and paclitaxel produced cell cycle arrest in the G2/M phase, while, in contrast, esculetin did not. These results could be relevant to designing new combined therapies against resistant prostate cancer.

## 1. Introduction

Prostate cancer is a serious disease with a growing incidence, for which no efficient treatment exists. The metastatic type is one of the most therapy-resistant human cancers. The primary treatment for this cancer is androgen ablation [1,2]. However, most patients develop androgen-independent tumours following androgen therapy. PC-3 prostatic tumour cells do not express androgen receptors and constitute one of the most commonly used prostate cancer cell lines. This cell line, established from bone-derived metastasis, represents an aggressive phase of cancer [3]. Moreover, it is an excellent experimental model for studying new therapies for human prostate carcinomas [4,5]. The current work was carried out using this single cell line because of its responsiveness to glucocorticoids, androgens and fibroblast growth factors. Other cell lines such as DU145, although hormone-insensitive, are less metastatic than PC-3 cells. Prostatic cell lines such as LNCaP show variable sensitivity to androgens depending on the subline used (LNCaP-G4 with high sensitivity; LNCaP-E9 with low sensitivity).

Paclitaxel and vinblastine are two chemotherapeutic drugs used in treatments for this type of cancer due to their target being tubulin.

Paclitaxel belongs to the taxane family, which shows antitumour activity on different cell lines representative of several haematological diseases [6]. It is also active on solid tumours such as those affecting the ovaries, breasts and lungs [7]. Paclitaxel acts by promoting microtubule assembly [8,9] and interferes with several signal transduction pathways, inducing subsequent apoptosis [10].

Vinblastine and other structurally related alkaloid compounds are used extensively as antitumour drugs. They bind tubulin, and they can be applied in therapy for prostatic tumours [11,12,13].

However, both paclitaxel and vinblastine have shown limited therapeutic efficacy and induction of toxicity, as well as multiple drug resistance. The current dilemma of using either of these antitumour compounds is that neither docetaxel nor vinblastine alone provide long-term control for castration-resistant prostate cancer. Their effectiveness diminishes as cancer cells develop resistance. Once a patient’s cancer has acquired resistance to docetaxel, the possibilities of treatment become limited. Therefore, further studies are required for a deeper knowledge of new therapeutic approaches.

Esculetin (6,7-dihydroxycoumarin) is a derivative of coumarin present in chicory and in many plants, both toxic and medicinal [14,15]. It is a natural lactone derived from the intramolecular cyclisation of a cinnamic acid derivative. The antioxidant properties of esculetin are considered beneficial. Esculetin has been found to modulate cyclooxygenase and inducible nitric oxide synthase [16,17,18,19,20], as well as redox balance in diverse cell types [18]. At low doses (1–25 µM), it has been demonstrated to suppress H_2_O_2_-induced cell damage in human hepatoma HepG2 cells [21]. In contrast, at higher doses (100–1000 µM), esculetin induces apoptosis in several cell lines such as adipocyte 3T3-L1 cells [22], human leukaemia U937 cells [23,24] and human promyelocytic leukaemia HL-60 cells [25]. Previous studies by our group have shown that esculetin is cytotoxic for NB4 human acute promyelocytic leukaemia cells by affecting redox balance [26,27,28,29].

Different drugs are useful for therapy in oncogenic processes of diverse origins, considering tissue cell specificity, sensitivity to hormones, metastatic proliferation, resistance acquisition, etc. Combined therapies using different antitumour drugs are now extensively used, having displayed high efficacy in several cases. For instance, esculetin enhanced taxol-induced apoptosis in hepatoma cells [30]. Additionally, several coumarins have been considered to reduce vinblastine resistance in MDCK-MDR1 cells [31]. By contrast, its antioxidant action can reduce cyclophosphamide-induced hepatotoxicity in vivo [32]. In this respect, some compounds can either increase or reduce the toxicity of other therapeutic drugs. Hence, we explored the toxic action of esculetin on PC-3 human prostate cancer cells which are insensitive to hormone therapy. We studied comparatively the cytotoxic effect of esculetin in relation to vinblastine and paclitaxel on PC-3 cells. These studies can be relevant for the application of combined drug therapies on tumour prostate cells. These results can open the possibility of combined treatments in order to increase antitumour effects or even to reduce the toxicity of current therapy treatments.

## 2. Materials and Methods

PC-3 cells obtained from the ATCC (American Type Cell Collection, Manassas, VA, USA; CRL-1435) were cultured for 24 h, prior to treatment, at 37 °C under a humidified atmosphere with 5% CO_2_ in RPMI medium 1640 with GlutaMAX™ supplemented with 10% heat-inactivated foetal bovine serum, 100 U/mL penicillin, 100 μg/mL streptomycin and 0.25 μg/mL amphotericin B [32].

### 2.1. Treatments

Cells were treated with either esculetin (100 or 250 μM), vinblastine (50 μM) or paclitaxel (100 or 200 μM) for 19 to 72 h. After incubation, cells were trypsinised, centrifuged at 300× *g* for 5 min and resuspended in phosphate-buffered saline (PBS).

### 2.2. Metabolic Activity

The MTT colorimetric assay [3-(4,5-dimethyl-2-thiazolyl)-2,5-diphenyltetrazolium bromide, Roche] was used to detect mitochondrial succinate dehydrogenase activity, hence giving an index of metabolic activity and cellular proliferation. After treatments, the cells seeded in 96-well microplates were incubated with 10 μL MTT Labelling Reagent for 4 h, and then 100 μL of a solubilisation solution was added. Tetrazolium salts are cleaved to formazan by the succinate–tetrazolium reductase system (EC 1.3.99.1), which belongs to the respiratory chain of mitochondria, and it is only active in metabolically intact cells. A microplate reader rendered measurements of absorbance which correlated with the number of metabolically viable cells.

### 2.3. Cytotoxicity

Cell death by necrosis was determined by measuring permeability to propidium iodide using flow cytometry. Propidium iodide (PI) is a standard reagent used to assess cell viability in terms of integrity. PI binds to double-stranded DNA but is excluded from cells with an intact plasma membrane. After treatments, 2.5 × 10^5^ cells were washed with 500 μL phosphate-buffered saline (PBS) and resuspended in 300 μL PBS. Then, 15 μL of 20 μg/mL PI solution (Calbiochem) was added, and the fluorescence was measured using a Becton Dickinson FACScalibur flow cytometer (San José, CA, USA).

### 2.4. Cell Cycle Analyses

Samples containing 3–5 × 10^5^ cells were incubated with 0.5 mg/mL RNase A for 30 min. They were then permeabilised with 0.1% Nonidet-P40 and incubated with 50 μg/mL propidium iodide. DNA content and cell cycle analyses were carried out by flow cytometry (FL-2 detector in a linear mode) using the software programs WinMDI 2.8 and Cylchred 1.0.2. Fluorescent signals were observed following excitation at 488 nm with a broad emission centred around 600 nm. Apoptotic cells were identified as hypodiploid peaks (sub-G_0_/G_1_ contents). Cells arrested at the G2/M cell cycle phase can be detected as an increase in the M4 peak of a fluorescence histogram.

### 2.5. Apoptosis Analyses by Annexin-V-FITC Cytometry Assay

Apoptosis was also measured by studying the presence of phosphatidylserine on the cell surfaces after double staining with annexin V-FITC (fluorescein isothiocyanate) and PI according to the manufacturer’s instructions (Annexin V-FITC Apoptosis Detection Kit; Calbiochem, San Diego, CA, USA), and analysed by flow cytometry (FACScan, Becton Dickinson, San José, CA, USA). After treatments, 2.5 × 10^5^ cells were centrifuged at 1200 rpm for 5 min and incubated with 500 μL of 1× Annexin V Binding Buffer and 1 μL of Annexin V-FITC for 5 min at room temperature in the dark. Then, 10 μL of PI were added and the apoptotic fraction was quantitated. Live cells correspond to the annexin V-negative and PI-negative (A^−^/PI^−^) quadrant. Since cells with sustained plasma membrane integrity do not take up PI, cells that are stained with annexin V but not with PI are in the early stages of apoptosis (A^+^/PI^−^). In late apoptosis or necrosis secondary to apoptosis, the cell membrane loses its integrity, allowing staining of the cells with both annexin V and PI (A^+^/PI^+^). Cells stained only with PI (A^−^/PI^+^) are considered necrotic. The results were analysed using WinMDI 2.8 software.

### 2.6. Statistical Analysis

The data are shown as the means ± standard errors of the means of at least three independent experiments. Treated samples were compared to control untreated cells using *t*-tests (one tail, heteroscedastic), and the *p*-values were reported in graphs using asterisks (*). Significant differences were considered to start at *p* = 0.05, with one, two or three asterisks indicating *p* < 0.05, *p* < 0.01 or *p* < 0.001, respectively. For assessing significance among different times of treatment, a one-way ANOVA was employed on groups of samples treated under the same conditions for increasing times (from 19 to 72 h).

## 3. Results

In this work, we studied the effect of esculetin in comparison with vinblastine and paclitaxel on PC-3 cells over time periods ranging from 19 to 72 h of treatment. Our previous studies showed that the lowest concentration of esculetin that induced a decrease in the viability of human leukaemia NB4 cells was 100 μM applied for 19 h [26]. Therefore, these conditions were used as a reference to begin our study on the influence of the antioxidant properties of esculetin on PC-3 prostatic tumour cell survival.

### 3.1. Metabolic Viability

**Figure 1:**
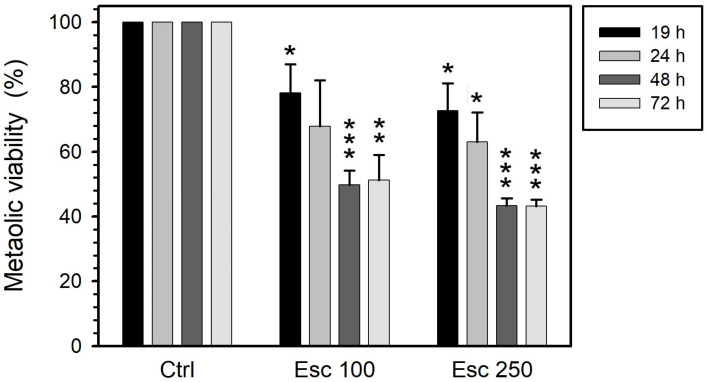
Metabolic activity of PC-3 cells treated with esculetin (Esc) at concentrations of either 100 µM or 250 µM for 19, 24, 48 or 72 h. We measured this parameter by MTT colorimetric assay to detect mitochondrial succinate dehydrogenase activity. Results present the means ± SEMs of at least three independent experiments. Asterisks (*) indicate comparisons between treated and control untreated samples (* *p* < 0.05, ** *p* < 0.01 and *** *p* < 0.001). Regarding the effect of the time of treatment under the same conditions, the decrease did not reach significance for 100 µM (*p* = 0.15) and was significant (*p* = 0.015) for 200 µM.

Esculetin decreased the metabolic activity of PC-3 cells to 70–80% when its concentration was increased from 100 to 250 µM at 19 h of treatment (Figure 1). This effect on metabolic activity progressively increased during the first 48 h. Treatments for more than 48 h had no further effect on metabolic activity, which remained at 50% for 100 µM esculetin and around 43% for 250 µM esculetin (Figure 1). All these reductions are significant with respect to control samples.

**Figure 2:**
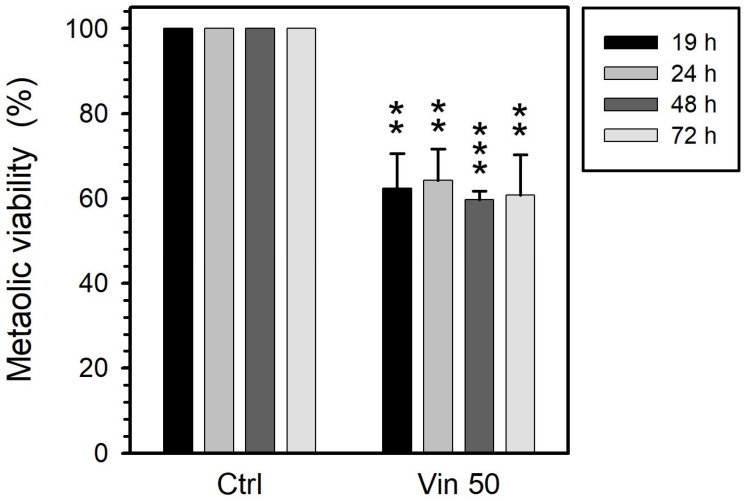
Metabolic activity of PC-3 cells treated with 50 μM vinblastine (Vin) for 19, 24, 48 or 72 h. This parameter was measured through MTT colorimetric assay, sensitive to mitochondrial succinate dehydrogenase activity. The results present the means ± SEMs of at least three independent experiments. Asterisks (*) indicate comparisons between treated and control untreated samples (** *p* < 0.01 and *** *p* < 0.001). Regarding the effect of the time of treatment, there were no significant differences.

At 19 h, vinblastine (50 µM) reduced metabolic activity by 40%. Longer treatments (up to 72 h) had no effect on this parameter (Figure 2). All the obtained results are statistically significant with respect to control experiments.

**Figure 3:**
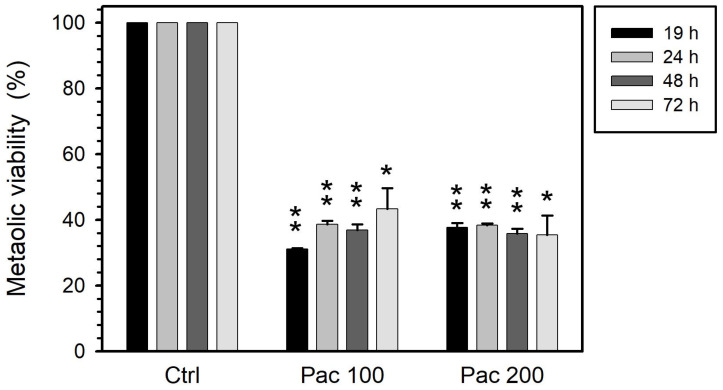
Metabolic activity of PC-3 cells treated with either 100 or 200 µM paclitaxel (Pac) for 19, 24, 48 or 72 h. We measured this parameter by MTT colorimetric assay to detect mitochondrial succinate dehydrogenase activity. The results represent the means ± SEMs of at least three independent experiments. Asterisks (*) indicate comparisons between treated and control untreated samples (* *p* < 0.05 and ** *p* < 0.01). Regarding the effect of the time of treatment under the same conditions, there were no significant differences.

Paclitaxel significantly reduced the metabolic activity of treated cells to 40%, an effect that was both concentration- and time-independent (Figure 3): Increasing paclitaxel concentration from 100 to 200 µM did not provoke a further reduction. Also, at both concentrations the extent of reduction achieved after 19 h did not progress with exposure up to 72 h.

### 3.2. Cell Viability

**Figure 4:**
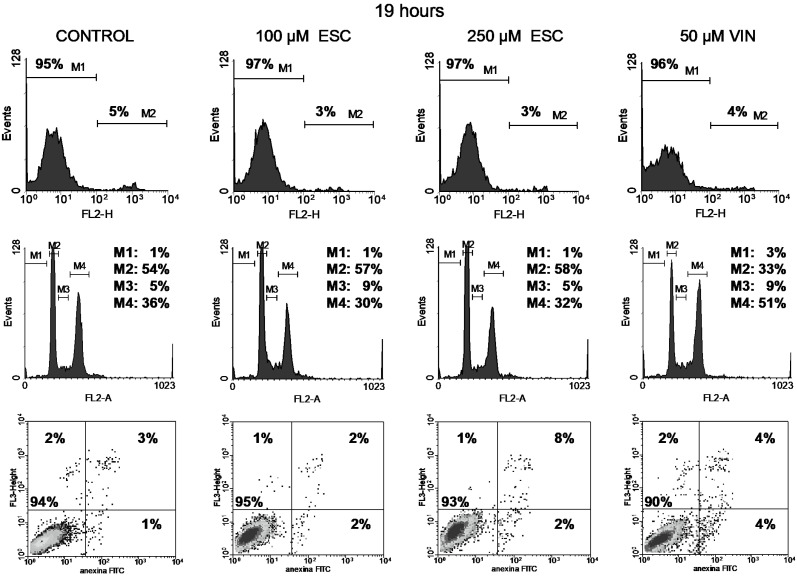
Cell viability with PI (top), cell cycle analysis (middle) and apoptosis with annexin V-FITC plus PI (bottom) of PC-3 cells treated for 19 h either with 100 or 250 µM esculetin (ESC) or 50 μM vinblastine (VIN). In the bottom panel, shades of black, gray and isolated dots reflect decreasing density of cell events.

**Figure 5:**
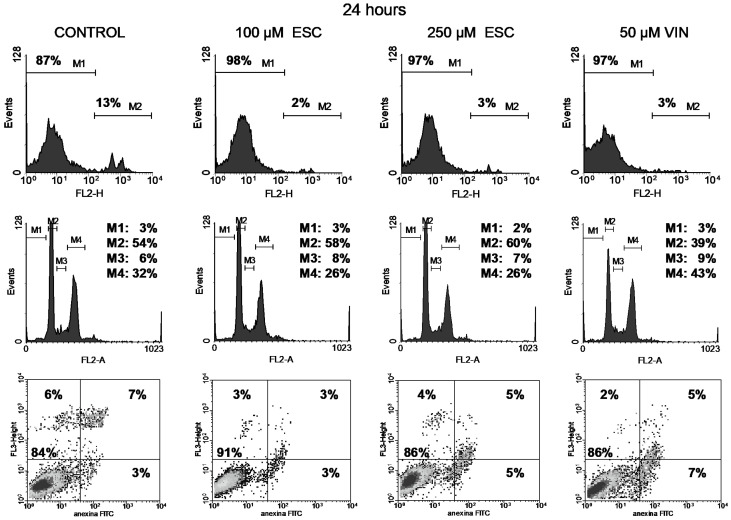
Cell viability with PI (top), cell cycle analysis (middle) and apoptosis with annexin V-FITC plus PI (bottom) of PC-3 cells treated for 24 h either with 100 or 250 µM esculetin (ESC) or 50 μM vinblastine (VIN). In the bottom panel, shades of black, gray and isolated dots reflect decreasing density of cell events.

**Figure 6:**
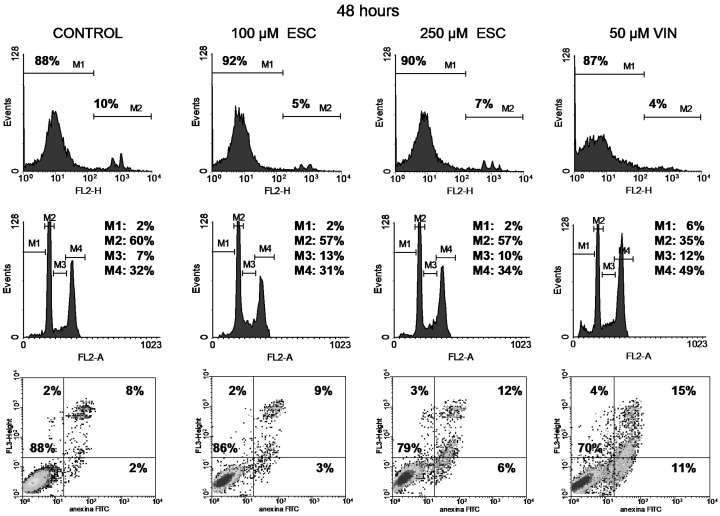
Cell viability with PI (top), cell cycle analysis (middle) and apoptosis with annexin V-FITC and PI (bottom) of PC-3 cells treated for 48 h either with 100 or 250 µM esculetin (ESC) or 50 μM vinblastine (VIN)**.** In the bottom panel, shades of black, gray and isolated dots reflect decreasing density of cell events.

**Figure 7:**
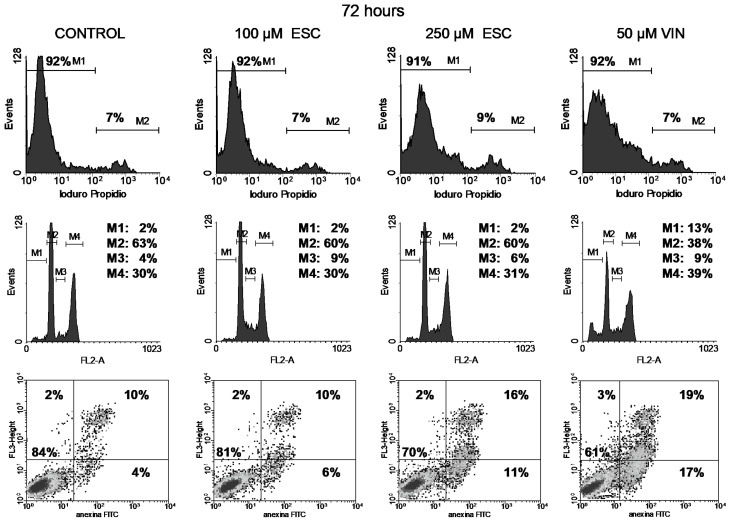
Cell viability with PI (top), cell cycle analysis (middle) and apoptosis with annexin V-FITC and PI (bottom) of PC-3 cells treated for 72 h either with 100 or 250 µM esculetin (ESC) or 50 μM vinblastine (VIN)**.** In the bottom panel, shades of black, gray and isolated dots reflect decreasing density of cell events.

Cell viability was studied through the permeability to propidium iodide (PI). The integrity of cell membranes revealed low cell damage after treatment (upper panels in Figure 4, Figure 5, Figure 6 and Figure 7). Esculetin (100 µM) did not affect PC-3 cell viability during the first 48 h as compared to the control. Even after the longest treatment (72 h) with the higher esculetin concentration (250 µM), the PI fluorescence pattern did not depart much from that of the controls. We observed similar results for vinblastine (Figure 4, Figure 5, Figure 6 and Figure 7).

**Figure 8:**
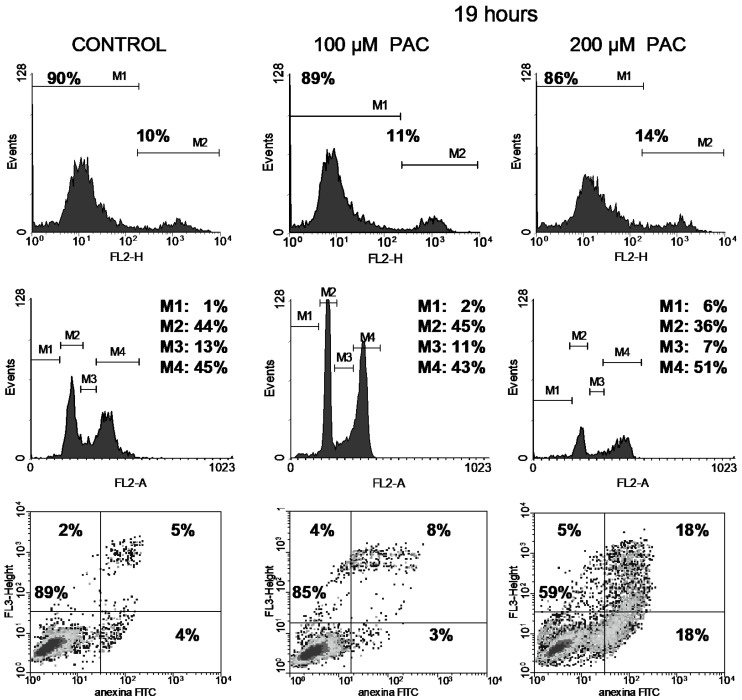
Cell viability with PI (top), cell cycle analysis (middle) and apoptosis with annexin V-FITC and PI (bottom) of PC-3 cells treated for 19 h either with 100 or 200 µM paclitaxel (PAC). In the bottom panel, shades of black, gray and isolated dots reflect decreasing density of cell events.

In contrast, treatment of cells with paclitaxel did impact viability somewhat, increasing the population of damaged cells from 10% to 14% for the 200 µM dose after the shortest 19h treatment (Figure 8).

### 3.3. Cell Cycle

With respect to the cell cycle, we can discern different patterns after treatments with these compounds.

Vinblastine interferes with the PC-3 cell cycle, as shown by an increment of the M4 peak (G2/M phase) since the shortest 19 h treatment (Figure 4, Figure 5, Figure 6 and Figure 7). This was accompanied by increased DNA fragmentation (increment of the M1 peak) perceived after 48 and 72 h exposure. Paclitaxel, at the highest concentration tested, also produced cell cycle arrest in the G2/M phase following 19 h of treatment, although to a lesser extent (Figure 8). This cell cycle arrest could be expected since vinblastine and paclitaxel are antimitotic compounds which interact with tubulin.

In contrast, esculetin did not significantly affect the cell cycle at any of the concentrations or time periods analysed (Figure 4, Figure 5, Figure 6 and Figure 7), since the proportions of the different fluorescent peaks in the diagrams remained similar for treatments with both concentrations of esculetin and all durations tested.

### 3.4. Apoptosis

Cells treated with the high esculetin concentration (250 µM) for both 48 and 72 h showed levels above 10% for early apoptosis, reaching 27% for total apoptosis (early plus late apoptosis) after 72 h of exposure.

The application of 50 µM vinblastine produced similar results (Figure 4, Figure 5, Figure 6 and Figure 7). The 48 h treatment rendered 15% early apoptotic cells plus 11% in late apoptosis. The longest (72 h) exposure raised early apoptosis to 19% and late apoptotic cells to 17%. These figures add up to 26% and 36% total apoptosis at 48 and 72 h.

On the other hand, paclitaxel at 200 µM led to high apoptosis (36% of total apoptosis) during the first 19 h of treatment (Figure 8).

## 4. Discussion

The main aim of this study was to analyse the toxic effects of esculetin, a coumarin with antioxidant action, on PC-3 human prostatic tumour cells. These cells do not express androgen receptors and hence constitute a hormone-independent model, and they are some of the most widely used tools in studying the action of chemotherapeutic drugs. Therefore, we aimed to analyse the induction of PC-3 cell death by some chemotherapeutic compounds.

Chemotherapeutic drugs such as paclitaxel, vincristine, vinblastine, etoposide, doxorubicin and camptothecin have demonstrated significant apoptosis induction in cancer cells through up-regulation of Bax and Bak and the induction of caspase activation, inhibition of angiogenesis, eradication of established tumours and enhancement of survival in mice [33].

Thus, toxicity and apoptosis induced by some drugs render reductions in neoplasms and increase organism survival. Different therapies can be tentatively suggested for metastatic prostate cancer. Antimitotic drugs such as taxanes or *Vinca* derivatives are antitumour compounds which have proven their efficacy in different human cancers.

Paclitaxel is a taxane compound that promotes microtubule polymerisation giving rise to cell death. Thus, paclitaxel can be used as an antitumour drug against cancer cells of different origins. For instance, paclitaxel induces DNA cleavage and cell death in NB4 leukaemia cells [34]. Paclitaxel also decreases p53 levels, changes in Bax and Bcl-2, and activation of caspase 3 and caspase 9 [34]. The induction of apoptosis by 0.1 nM paclitaxel was demonstrated after treatment for 24 h or longer [35]. When we treated PC-3 cells with paclitaxel (100 or 200 µM) for 19 h, we observed an induction of 36% apoptotic cells and an increase in permeability to PI (14% permeable cells).

Some taxane derivatives such as docetaxel are widely used in cancer therapy, though dosages are limited by haematological toxicity. In prostatic cancer, it is recommended to concomitantly use either prednisone or prednisolone. For instance, some cancer treatments have combined docetaxel with cisplatin, doxorubicin or capecitabin. Detailed considerations about the tolerability of these combined treatments on patients must be pursued [36,37].

We analysed the effects of vinblastine on human prostatic PC cells. At a concentration of 50 µM, it induced a 40% reduction in metabolic activity. Increasing the duration of treatment did not reduce this activity any further (Figure 2). Although treatments for 72 h induced the presence of 36% apoptotic cells (17% in early apoptosis plus 19% in late apoptosis), no relevant changes in cell membrane permeability to PI were observed, since 92% of the cells remained impermeable to propidium iodide (Figure 7, upper panel). Induction of apoptosis in PC-3 cells after 24 h treatment with vinblastine (62 µg/mL, 76 µM) has been reported by others [35].

We also used esculetin to treat PC-3 cells at two different concentrations (100 and 250 µM) for periods from 19 to 72 h. At the highest concentration and time, esculetin reduced metabolic viability to 45% of the initial activity. Cell cycle arrest was not observed, although apoptosis was induced (27%, adding the values for early and late apoptosis). Our results indicate a slight influence of the esculetin concentration on metabolic activity (Figure 1). In addition, we observed a reduced proportion of apoptotic and dead cells at 250 µM after 48 h (Figure 6), which is comparable to the results described for the application of 150 µM for 48 h in 3T3 cells [22]. Moreover, we did not find significant changes in the cell cycle profile produced by esculetin at any of the concentrations (Figure 4, Figure 5, Figure 6 and Figure 7).

These results are relevant for considering possibilities of combined drug therapy. In malignant melanomas, the combinations of esculetin with cisplatin, docetaxel and paclitaxel have demonstrated additive interactions [38]. Furthermore, esculetin enhances paclitaxel-induced apoptosis in human hepatoma [30]. The antitumour action of esculetin proceeds in cancer prostate cell lines through inhibition of cell proliferation and G_1_ phase cell cycle arrest [37]. Induced expression of p53, p21 and p27 and down-regulation of CDK2 and CDK4 could mediate antitumour esculetin action on prostate tumour cells [39]. Similar mechanisms of esculetin action are present in cases of human colon cancer [40], human breast cancer [41] and human leukaemia cells [42]. CDK2 and CDK4 are involved in the toxic action of paclitaxel on human hepatoma cells [43]. Furthermore, changes in NFκB mediated paclitaxel apoptosis in human leukaemia cells [34].

A relevant consideration in the use of esculetin either alone or in combination with antitumour drugs such as paclitaxel or vinblastine is the toxicity of this antioxidant itself.

Esculetin, because of its antioxidant properties, can act as a redox balance modulator. Thus, it showed protective effects against oxidants such as H_2_O_2_, even in cancer cells like leukaemia cells [44].

Obviously, both the antioxidant protective effect and the toxic action that will be relevant for antitumour use might be dependent on the concentration of the coumarin. Thus, when considering a coumarin as an alternative antitumour compound or as a reducer of the toxic action of other anticancer drugs, the concentration used must be consistently considered. The bioavailability of esculetin in pharmacokinetics is also a limiting factor to be kept in mind.

Oral bioavailability has been demonstrated for coumarin derivatives as antagonists for treating prostate cancer [45]. Anyhow, esculetin has shown both in vivo and in vitro protective effects., i.e., esculetin against isoproterenol induced myocardial infarction by antioxidant and myocardial membrane stabilisation, along with in vitro protection from arsenic-induced ROS cell necrosis or apoptosis in H9C2 cells [46]. Also, esculetin shows protective effects against the hepatotoxicity produced by some drugs [47], as well as the toxicity provoked by doxorubicin in PC-3 cells [48]. From another viewpoint, esculetin is efficient in vivo in liver cancer treatment [49].

The results shown in this work indicate that esculetin has a less toxic effect on PC-3 human prostate cancer cells. Although paclitaxel and vinblastine act on cell division rates, redox imbalance might be produced, and, tentatively, esculetin could protect against the toxic action of those antimitotic drugs on healthy cells. By contrast, additional toxic effects could be produced by esculetin in prostate cells treated with these antimitotic drugs, producing higher efficacy in therapy treatments. To study the in vivo action of a combination of esculetin with either paclitaxel or vinblastine, animal models should be developed and assayed.

These results add new knowledge on the therapeutic applications of esculetin [50,51,52,53] and could support the eventual application of therapies combining esculetin and antimitotic drugs. Additive interactions could be effective in therapies applied to tumour prostate cells.

## 5. Conclusions

Esculetin decreases the metabolic activity of PC-3 cells in a time- and concentration- dependent manner, whereas the impact on metabolic activity of vinblastine and paclitaxel is not time-dependent. This might make it feasible to control the action of esculetin on human cancer prostate treatments.

PC-3 cells treated with a high concentration (250 µM) of esculetin for 48 or 72 h show apoptosis levels slightly lower than those produced by 50 µM vinblastine at these incubation times or by 200 µM paclitaxel at 19 h. Thus, expectations of similar effects in vivo could be considered using these different concentrations of these compounds, which might be useful for combined therapies.

Vinblastine and paclitaxel produced cell cycle arrest in the G2/M phase after incubation for 19 h. Esculetin does not seem to significantly affect the cell cycle. Different mechanisms of action on PC-3 cells by these different compounds were confirmed.

The concentration-dependent action of esculetin plus its potential beneficial or toxic effects could be relevant in considering the combined use of these three different drugs in the treatment of human prostate hormone-independent cancers.

## Data Availability

The original data presented in the study are available at FigShare, https://doi.org/10.6084/m9.figshare.26274268 (accessed on 1 July 2025).
